# The evolution of diverse antimicrobial responses in vancomycin-intermediate *Staphylococcus aureus* and its therapeutic implications

**DOI:** 10.1101/2023.11.30.569373

**Published:** 2023-12-01

**Authors:** Dena Crozier, Jason Gray, Jeff A. Maltas, Robert A. Bonomo, Kyle J. Card, Jacob G. Scott

**Affiliations:** 1 Cleveland Clinic Lerner College of Medicine, Case Western Reserve University, Cleveland, OH; 2 Department of Medicine, Washington University School of Medicine, St. Louis, MO; 3 Department of Physics, Case Western Reserve University, Cleveland, OH; 4 Department of Translational Hematology and Oncology Research, Cleveland Clinic Lerner Research Institute, Cleveland, OH; 5 Department of Medicine, Louis Stokes Cleveland Department of Veteran Affairs Medical Center, Cleveland, OH

## Abstract

*Staphylococcus aureus* causes serious clinical infections, including bacteremia and endocarditis. When clinicians suspect these diseases, they prescribe broad-spectrum antibiotics like vancomycin and then adjust treatment based on antimicrobial susceptibility test results. However, these results reflect the infection’s state before treatment and do not account for any potential changes in its antibiotic response arising from intervening evolution. Thus, an initial test may indicate that the infection is susceptible to a particular drug, but this recommendation may be tentative. In this study, we aimed to understand how treatment effectiveness changes over time by accounting for evolution. First, we evolved 18 methicillin-susceptible *S. aureus* (MSSA) populations under increasing vancomycin concentrations until they reached intermediate resistance levels. We then sequenced their complete genomes to characterize the mutational paths to their evolved vancomycin-intermediate states. Lastly, we subjected these evolved populations to seven antibiotics used to treat MSSA infections and compared drug susceptibilities between the evolved lines and their common ancestor. Mutations in several genes responsible for regulating the cell membrane stress response and cell-wall biosynthesis appeared in parallel among the evolved vancomycin-intermediate *S. aureus* (VISA) populations, and these lines were repeatedly cross-resistant to daptomycin. These observations align with previous clinical findings, reinforcing the role of these genes in mediating resistance. In contrast, the populations exhibited diverse responses to other antibiotics. Most lines were susceptible to meropenem, gentamicin, and nafcillin, but several replicates were also resistant. We accounted for this diversity by deriving likelihood estimates that express a population’s probability of exhibiting a drug response following vancomycin treatment. Our findings support using antistaphylococcal penicillins (e.g., nafcillin) as a first-line treatment for MSSA, even in light of intermediate vancomycin resistance. They also emphasize the inherent uncertainty and risk that evolution poses to effective therapies, attributes one cannot account for by susceptibility tests alone. Instead, clinicians must consider infections as evolving systems that may take several different paths, with implications on their potential sensitivities to other drugs.

## Introduction

*Staphylococcus aureus* is a Gram-positive pathogen that poses a significant public health challenge because it causes diverse clinical infections.^1–4^ It enters the bloodstream through various means, often via a wound, surgical site, or catheter, to cause bacteremia.^1,3^ The infection may then progress to endocarditis when *S. aureus* attaches to the heart’s valves or inner lining, damaging these structures and disrupting normal blood flow.^5^ Patients with prosthetic valves, congenital heart conditions, or a history of intravenous drug use are at higher risk of contracting this disease.^4,6^ Due to their severity, clinicians initially treat these infections with broad-spectrum antibiotics. These empiric therapies often include vancomycin because it is effective against Gram-positive pathogens, including methicillin-resistant *S. aureus* (MRSA). Therapy is later narrowed based on the identified organism and its susceptibility profile. Antistaphylococcal penicillins, including nafcillin and oxacillin, are currently acknowledged treatments for methicillin-susceptible *S. aureus* (MSSA) infections. Nevertheless, we ask whether favorable susceptibility results alone justify their use. This question rests on two key considerations.

Firstly, the evolution of vancomycin-intermediate resistance during empiric treatment may affect *S. aureus*’ susceptibility to other drugs. Intermediate resistance evolves via mutations in a diverse set of genes^7–10^ and is associated with persistent infection and reduced treatment success.^10^ These mutations may alter MSSA’s response to subsequent first-line therapies despite having no prior exposure. For example, a large cohort study showed that the in-hospital mortality rate of cloxacillin-treated patients correlated with vancomycin minimum inhibitory concentration (MIC).^11^ MSSA should be susceptible to both cloxacillin and methicillin because these drugs have the same cellular target, and thus, prior vancomycin adaptation might explain these poor clinical outcomes.

Secondly, at best, antibiotic susceptibility test results are a lagging indicator of phenotypic state and agnostic about evolution. In current practice, clinicians sample a patient’s blood and then administer broad-spectrum antibiotics, isolate the causative agent of the infection from the blood cultures, and then rely on susceptibility tests to inform subsequent therapy. However, these tests provide information about the pathogen’s susceptibilities before treatment and, thus, cannot account for any potential changes in its antibiotic response arising from intervening evolution. Therefore, an initial test may indicate that the infection is susceptible to an antistaphylococcal penicillin, but this recommendation may be tentative.

In this study, we examined how treatment effectiveness changes over time by accounting for evolution. Ideally, a regimen for MSSA would include drugs the infection has a high chance of being susceptible to, given its prior exposure to empiric vancomycin therapy. To address these issues, we compared the phenotypic and genotypic responses of evolved vancomycin-intermediate *S. aureus* (VISA) populations against a common ancestor. Mutations in cell membrane stress response and cell-wall synthesis genes appeared in parallel across the evolved VISA populations, and these lines were repeatedly cross-resistant to daptomycin. These observations align with previous findings,^12,13^ reinforcing the role of these genes in mediating resistance.

Conversely, the populations exhibited variable responses to other antibiotics. While most were susceptible to meropenem, gentamicin, and nafcillin, some were resistant. We addressed this variability by estimating the likelihood that a population expresses a particular drug response after vancomycin treatment. Our data support the first-line use of antistaphylococcal penicillins like nafcillin for treating MSSA, even when intermediate vancomycin resistance is present. They also highlight the uncertainty and risk of not accounting for evolution when making therapeutic decisions. Instead, clinicians should anticipate that infections will evolve under empiric therapy, which might affect their sensitivities to first-line drug treatment. Our study, therefore, underscores the complexities of bacterial evolution and offers a more dynamic, data-driven approach to antibiotic treatment.

## Results and Discussion

### Evolution of vancomycin-intermediate resistance in MSSA

**Figure 1.**
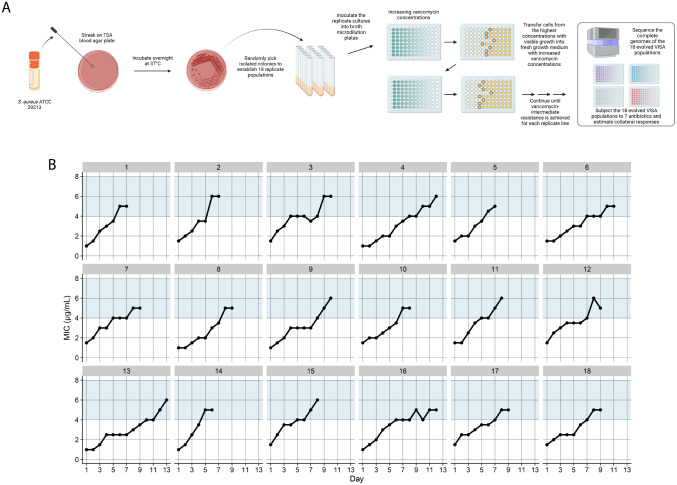
Schematic illustration of our study design and evolution of intermediate vancomycin resistance. (A) We established 18 independent populations from the methicillin-susceptible *S. aureus* (MSSA) strain and experimentally evolved them with increasing vancomycin concentrations until they reached intermediate resistance levels. We then performed whole-genome sequencing on these evolved populations and examined their susceptibilities to seven drugs used in MSSA treatment. (B) By day 13 of the evolution experiment, all populations evolved intermediate resistance, defined by the Clinical and Laboratory Standards Institute (CLSI) as a minimum inhibitory concentration (MIC) between 4 and 8 μg/mL (blue-shaded regions).

First, we established 18 replicate MSSA populations from an ancestral clone of *S. aureus* subsp. *aureus* Rosenbach, a clinical isolate that is an international quality control standard. We transferred each population daily to a growth medium with vancomycin and progressively increased its concentration until the lines reached intermediate resistance levels (Methods) (Figure 1A). The Cleveland Clinic Institutional Biosafety Committee approved all methods. The lines had an initial average MIC of 1.3 μg/mL and evolved to an average MIC of 5.4 μg/mL in 9 days (Figure 1B).

### Genomic analysis reveals mutations and pathways in vancomycin-intermediate resistance

Next, we sequenced the ancestor and experimentally-evolved populations to investigate the genetic changes responsible for vancomycin-intermediate resistance. The lines had a total of 102 mutations. However, we curated the sequencing results by excluding mutations in multicopy elements and several other criteria (Methods). We then evaluated the extent of genomic parallelism in these lines by focusing only on the 88 curated mutations. We found that the average between-line similarity was 0.11. In other words, on average, two VISA populations had 11% of their mutated genes in common, and most of the mutations only occurred in a single line.

**Figure 2.**
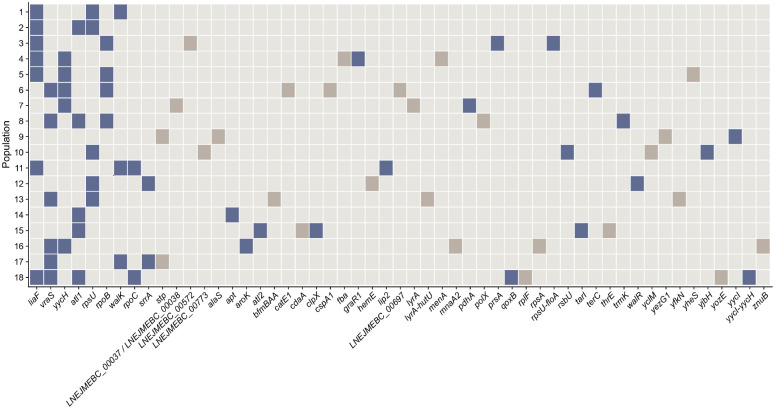
Identity of mutated genes in the intermediate vancomycin-resistant lines. Filled cells represent the 88 curated mutations (Methods) that evolved in the 18 methicillin-susceptible *S. aureus* populations during vancomycin exposure. The blue-shaded cells identify mutations that have been previously observed in laboratory and clinical isolates.^14–24,27–41^ We list the affected genes along the bottom in order of the total number of mutations.

Nonetheless, several mutations repeatedly appeared and are likely associated with increased vancomycin MICs (Figure 2). First, mutations in *liaF* occurred most frequently in 7/18 (38.9%) lines. Given a genome size of 2,586 coding sequences and assuming each gene has an equal length,^14^ the probability that any gene is mutated seven times is 3.3 × 10^−21^. Second, 6/18 (33.3%) lines had mutations in *vraS* (*P* = 8.6 × 10^−18^). LiaF and VraS regulate the cell membrane stress response to organic solvents, detergents, and lipid II cycle inhibitors, including vancomycin.^15,16^ Third, six populations had mutations in either *rpoB* or *rpoC*. These genes encode the *β* and *β*^′^ subunits of the RNA polymerase, respectively. Mutations in these genes are associated with vancomycin-intermediate resistance in laboratory^17,18^ and clinical *S. aureus* isolates.^19^ Fourth, the *yycH* and *walK* genes comprise a two-component regulatory system in *S. aureus* involved in cell-wall metabolism and vancomycin resistance.^20^ Moreover, the downregulation of *walKR* increases virulence by decreasing the release of pathogen-associated molecular patterns (PAMPs) that trigger the host immune response.^21^ These genes were mutated in five (*P* = 2.2 × 10^−14^) and three (*P* = 1.5 × 10^−7^) lines, respectively. Fifth, five out of 18 populations had mutations in *atl* (*P* = 2.2 × 10^−14^). This gene encodes a bifunctional autolysin that separates daughter cells and acts as an adhesin by binding to fibronectin and vitronectin.^22,23^ Atl is also involved in biofilm formation, but this phenotype depends on methicillin resistance.^24^ For example, deleting *atl* decreased biofilm production in MRSA clinical isolates but not in methicillin-susceptible backgrounds.^24^

These findings align with clinical observations. Hafer et al. showed that several nonsynonymous point mutations in *vraRS*, *walRK*, and *rpoB* were prevalent in clinical VISA isolates but not in vancomycin-susceptible *S. aureus* (VSSA), suggesting an association with decreased susceptibility.^25^ Their results and ours suggest that the vancomycin-intermediate resistance phenotype evolves via mutations in diverse pathways. Moreover, *S. aureus* sometimes evolves *atl* mutations that reduce cell lysis caused by vancomycin exposure. This phenotype prolongs drug survival and promotes the evolution of secondary mutations that cause vancomycin-intermediate resistance in clinical strains.^26^

### Collateral resistance and sensitivity in VISA populations and their implications for treatment

Then, we examined how evolution under vancomycin selection affected the VISA populations’ susceptibility to other antibiotics. These so-called collateral drug responses widely occur in bacteria^2,42–49^ and cancer,^50–52^ and may affect therapeutic outcomes. We compared the MICs of the ancestral clone against the 18 evolved populations in cefazolin, clindamycin, daptomycin, gentamicin, meropenem, nafcillin, and trimethoprim-sulfamethoxazole. These antibiotics are suggested treatments for MSSA bacteremia^53^ and endocarditis.^2,54^ They also have diverse mechanisms of action: cefazolin, meropenem, and nafcillin inhibit cell-wall biosynthesis; clindamycin and gentamicin interfere with protein synthesis by binding to the 50S and 30S ribosomal subunits, respectively; daptomycin disrupts cell-membrane function; and trimethoprim-sulfamethoxazole inhibits folate biosynthesis, thus preventing DNA replication. For each antibiotic, we quantified the collateral response of an evolved population as the difference in its log_2_-transformed MIC relative to the ancestral clone. A population is collaterally resistant when its MIC is higher than the ancestral MIC and collaterally sensitive when it is lower.

**Figure 3.**
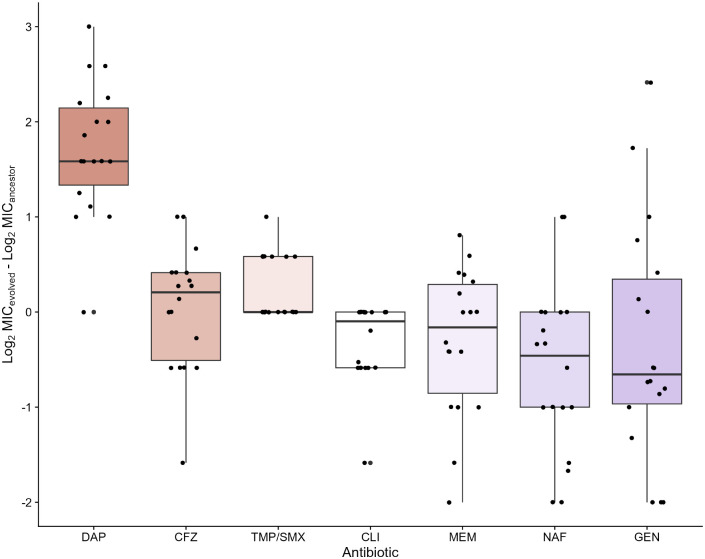
Collateral responses vary to several first-line drugs. The median and interquartile range of collateral response values are shown for daptomycin (DAP), cefazolin (CFZ), trimethoprim-sulfamethoxazole (TMP/SMX), clindamycin (CLI), meropenem (MEM), nafcillin (NAF), and gentamicin (GEN). For each antibiotic, the collateral response of an evolved vancomycin-intermediate resistant line was calculated as the difference between its log_2_-transformed minimum inhibitory concentration (MIC) and the log_2_-transformed MIC of the ancestor. Points represent the average collateral drug responses among 3 technical replicates for each of the 18 populations. We order the seven antibiotics by decreasing median collateral resistance.

There were robust collateral responses to several antibiotics. Seventeen out of 18 VISA populations (94.4%) exhibited increased daptomycin resistance (*U* = 4, *p* < 0.001) (Figure 3), and nine lines had MICs above the clinical breakpoint for this phenotype.^55^ Thus, the replicates evolved intermediate vancomycin resistance and, as a result, became resistant to daptomycin despite never being exposed to this drug. Bacteria evolve thicker cell walls under vancomycin selection that hinder daptomycin entry,^12,13^ possibly explaining our results and clinical accounts of collateral resistance between these drugs in *S. aureus* isolates.^56^ Given our findings, we emphasize caution in treating methicillin-susceptible VISA infections with daptomycin. Our recommendation mirrors the 2023 European Society of Cardiology guidelines for endocarditis treatment, which state that clinicians should administer this drug with another antistaphylococcal antibiotic to lower the chances of treatment failure.^54^ Moreover, although clinical outcomes are similar between MSSA bacteremia patients treated with daptomycin and antistaphylococcal penicillins,^57^ clinicians should consider the increased risk associated with daptomycin cross-resistance.

The VISA lines also consistently responded to trimethoprim-sulfamethoxazole and clindamycin. Six of the 18 populations (33.3%) were resistant to trimethoprim-sulfamethoxazole (*U* = 44, *p* = 0.044), and 9/18 were sensitive to clindamycin (*U* = 108, *p* = 0.018) (Figure 3). In both cases, the remaining populations exhibited no change in MIC relative to the ancestor. Although the clindamycin results appear promising, monotherapy is associated with higher rates of recurrent infection in MSSA endocarditis because this drug inhibits the reproduction of bacteria without killing them.^58^ Nonetheless, these drugs are effective when given together.^59^

The evolved populations exhibited diverse responses to the remaining antibiotics, with no significant changes in either direction. Most lines were susceptible to meropenem, gentamicin, and nafcillin, but several replicates were also resistant. For example, nine and 11 lines were more sensitive to meropenem and gentamicin than the ancestor, respectively, and six lines were more resistant in both cases (Figure 3). However, in no instance did a line’s MIC meet or exceed the clinical breakpoint for resistance.^55^ Additionally, only two populations evolved collateral resistance to nafcillin, whereas 12 populations evolved increased sensitivity.

Nafcillin and other antistaphylococcal penicillins are the current recommended treatments for MSSA infections. Nonetheless, some clinicians support first-line cefazolin therapy instead because patients can take this drug orally and tolerate its side effects better.^60–63^ These attributes improve medication adherence. Other clinicians prefer nafcillin because cefazolin is susceptible to cleavage by *S. aureus*-produced penicillinases and, therefore, may be less effective when there is a high bacterial titer.^64,65^ Our results offer an evolutionary perspective on this debate. We showed that evolution under vancomycin selection caused increased cefazolin resistance in many replicate populations: 10 lines were more resistant than the ancestor, whereas six were more sensitive (Figure 3). An ideal first-line regimen for MSSA should include drugs to which the infection has the highest chance of being collaterally sensitive. Therefore, we recommend nafcillin over cefazolin based on an evolutionary consideration.

Drug-response outcomes might change, however, in different experimental and clinical contexts. Here, we outline two possible future studies that address these contexts and generalize our findings. First, one could examine whether *S. aureus* clinical isolates with different genetic backgrounds exhibit similar collateral sensitivity or resistance patterns. A bacterium’s background may direct evolution toward some pathways while constraining others.^66,67^ This process, called historical contingency, may affect how well a population evolves resistance^39,68–70^ and its responses to other drugs. For example, two studies analyzed the repeatability in drug responses of independently derived *Escherichia coli* or *Enterococcus faecalis* populations.^48,49^ Both found that the populations accumulated genetic differences as they evolved resistance to the same antibiotic, and these differences influenced their reaction to other drugs in sometimes unpredictable ways. Second, one might investigate how biofilm formation impacts collateral drug responses in MSSA and how different antibiotic delivery systems alter these patterns. These studies would bridge the gap between laboratory findings and clinical applicability, especially considering the relevance to implant-associated infections caused by biofilms and heart valve vegetations.^71,72^

### Prospects of using likelihood estimates to inform antibiotic treatment

When clinicians use antimicrobial susceptibility test results to guide treatment choices, they participate in a decision-making process with inherent uncertainty and risk. For example, they base their decisions on the available information, but it may not always be the most accurate or current. Instead, the results might reflect the pathogen’s susceptibilities before empiric therapy, ignoring any adaptations the bacteria might evolve in response to treatment. Our findings highlight this possibility. The evolution of intermediate vancomycin resistance in MSSA diversely affected its susceptibilities to meropenem, nafcillin, and gentamicin, which are first-line drugs used to treat endocarditis. Thus, confidence in the accuracy and relevance of initial susceptibility test results may lead to suboptimal treatment choices.

**Figure 4.**
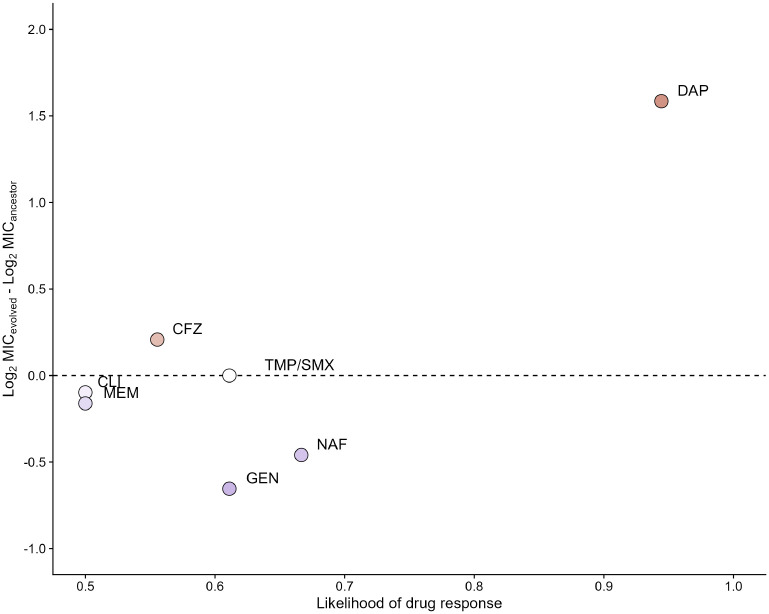
Likelihood that a population will exhibit a drug response following vancomycin selection. Points are shaded by the populations’ median drug response, calculated as the difference between an evolved line’s log_2_-transformed minimum inhibitory concentration (MIC) and the log_2_-transformed MIC of the ancestor in cefazolin (CFZ), clindamycin (CLI), daptomycin (DAP), gentamicin (GEN), meropenem (MEM), nafcillin (NAF), and trimethoprim-sulfamethoxazole (TMP/SMX). The horizontal line represents no change in response, whereas points above or below the line denote that the lines were collaterally resistant or sensitive overall. Likelihoods represent the proportion of measurements that align with the median response (Methods).

To address this concern, we propose a likelihood estimate that expresses a population’s probability of exhibiting a drug response following empiric treatment. We computed this estimate for each antibiotic by comparing the MIC measurements of the evolved populations to the median MIC (Methods). The VISA populations were more consistently susceptible to nafcillin than gentamicin or meropenem: a population had a 66.7% chance of being nafcillin-susceptible, whereas only a 61.1% and 50.0% chance of being gentamicin- or meropenem-susceptible, respectively (Figure 4). This analysis supports our recommendation for using nafcillin as a first-line antibiotic to treat MSSA bacteremia and endocarditis.

Likelihood estimates provide a way for clinicians to assess the probable effectiveness of different first-line drugs based on a population’s prior evolutionary history. Incorporating these estimates and a broader evolutionary perspective into clinical protocols will guide healthcare providers in making more informed treatment decisions. The ability to predict enables a strategic approach to antibiotic selection that could reduce the incidence of treatment failures and the selection of resistant strains. This shift toward evolutionary-informed therapy represents an important step in combating antibiotic resistance.

## Methods

### Ancestral strain and experimental evolution

We used *Staphylococcus aureus* subsp. *aureus* Rosenbach (ATCC 29213) as the ancestor of our evolution experiment. This methicillin-susceptible strain was originally isolated from human pleural fluid in 1884^73^ and is an international quality control standard with defined susceptibilities to many antibiotics. According to the Clinical and Laboratory Standards Institute (CLSI)^55^, vancomycin’s MIC on this strain is 0.5 – 2 *μ*g/mL. Clinical *S. aureus* isolates with MICs <2 *μ*g/mL are therefore considered susceptible to this drug. The clinical MIC breakpoints differentiating intermediate- and complete-resistant phenotypes are 4 – 8 *μ*g/mL and >16 *μ*g/mL, respectively. We used these breakpoints to classify phenotypes in our study.

All experiments were performed at 37°C unless noted otherwise. We revived the ATCC 29213 strain from a frozen stock by streaking cells onto tryptic soy agar (TSA) plates supplemented with 5% sheep blood (Remel, Lenexa, KS). We randomly picked isolated colonies from these plates to establish 18 replicate populations in tryptic soy broth (TSB) (Cleveland Clinic, Cleveland, OH), ensuring that evolution in each population originated from independent mutational events.

To start the evolution experiment, we prepared a series of vancomycin dilutions in TSB, ranging in concentrations from 0.25 – 1.5 *μ*g/mL. Each replicate was aliquoted into equal volumes of the vancomycin-amended TSB for a total dilution of 1:400 and incubated for 22 hours. Next, we transferred 1:400 cells from the highest concentration with visible growth for each replicate into fresh TSB with increased vancomycin concentrations. We transferred the replicate populations every 18 – 24 hours until they grew at concentrations between 4 – 8 *μ*g/mL for two consecutive transfers. Replicates that did not grow at any concentration after 24 hours were left to incubate until they did so, but no longer than 96 hours. Undiluted cultures of the evolved populations were frozen at −80°C in TSB medium supplemented with 15% glycerol as a cryoprotectant.

### Whole-genome sequencing

Glycerol stocks of frozen ancestral and evolved-population samples were grown overnight in TSB with 1.5 *μ*g/mL vancomycin. We centrifuged the overnight cultures at 8,000 rpm for 3 minutes and removed the supernatant from the bacterial pellets. SeqCenter (Pittsburgh, PA) prepared sample libraries from these pellets using the Illumina DNA Prep kit and IDT 10bp UDI indices and sequenced them on an Illumina NextSeq 2000. Demultiplexing, quality control, and adapter trimming were performed with bcl-convert (v3.9.3). The resulting FASTQ files of 151-base paired-end reads were deposited to the NCBI Sequence Read Archive (accession no. xxx).

### Mutation identification and tests of genomic parallelism

The sequencing reads were filtered to remove low-quality bases using Trimmomatic v0.39.^74^ We clipped the reads when the average quality score was <20 in a 4-bp window and to a minimum length of 36 bp. Next, we used *breseq* v0.38.1^75^ to identify mutations in two steps. First, we used this computational pipeline with default parameters to map the ancestral strain reads to the ATCC 29213 reference genome. This step accounted for mutations present at the beginning of our evolution experiment. Second, we applied these background mutations to the ATCC 29213 genome and reran *breseq* in polymorphism mode to map the evolved-population reads to this updated reference.

We quantified the extent of parallelism in genomic evolution by comparing the gene-level similarity of mutations between the 18 independent VISA populations. Following the methods outlined in Deatherage et al.^76^ and Card et al.,^77^ we manually curated the sequencing results by excluding mutations below 15% frequency and within multicopy elements, such as ribosomal RNA operons and synthetases responsible for charging tRNA. Gene conversions may cause these mutations, but short-read sequencing data cannot fully resolve them.^77^ Moreover, we included only those mutations that impact a single gene. These include nonsynonymous point mutations, small indels that occur within genes, and mutations within 150 bp upstream of the start of a gene. Lastly, we included large deletions if at least one affected gene was mutated in another population. A total of 88 mutations remained after these curation steps.

We then estimated Dice’s similarity coefficient, *S*, for each evolved population pair, where *S* = 2|*X* ∩*Y*|*/*(|*X*|+|*Y*|). Here, |*X*| and |*Y*| are the sets of genes with mutations in two evolved populations, and |*X* ∩*Y*| is the set of genes with mutations in both populations. *S*, therefore, ranges from 0, when the pair share no mutations in common, to 1, when both have mutations in the same set of genes.^76,77^ Lastly, we calculated the average of these coefficients for all evolved-population pairs. However, this analysis does not consider the probability that any particular gene will be mutated more than once across the independent populations (i.e., parallelism). To address this issue, we estimated this probability by sampling with replacement that gene *n* times, given a genome of *N* genes. In this calculation, we assume that each gene has an equal length and uniformity of mutation rate across the genome.^14^

### Estimating collateral drug responses and likelihoods

We estimated the collateral responses of the 18 evolved VISA populations to subsequent antibiotics using the broth microdilution method outlined by the CLSI.^78^ We supplemented Mueller-Hinton broth (MHB) (Fisher Bioreagents, Ottawa, CA) with 20 – 25 mg/L Ca^2+^ (CaCl_2_ * 2H_2_O, Fisher Bioreagents, Ottawa, CA) and 10 – 15 mg/L Mg^2+^ (MgCl_2_, Invitrogen, Vilnius, LT). We used this cation-adjusted MHB (CA-MHB) to test the susceptibilities of the evolved populations to cefazolin, clindamycin, daptomycin, gentamycin, meropenem, nafcillin, and trimethoprim/sulfamethoxazole. For daptomycin and nafcillin susceptibility tests, we used CA-MHB that was additionally supplemented with 25 mg/L Ca^2+^ (for a total calcium concentration of 50 mg/L) and 2% (w/v) NaCl (Fisher Bioreagents, Ottawa, CA), respectively.^79^ CA-MHB was stored at 4°C to prevent ion precipitation.

We inoculated 100 *μ*L of cells from the frozen evolved-population samples into CA-MHB with 1 *μ*g/mL vancomycin (i.e., the MIC of the ancestral strain) to maintain resistance. After overnight growth, the cultures were diluted to a McFarland 0.5 standard and then again by 200-fold in MHB. We aliquoted an equal volume of these cells across a series of linear concentrations of a given antibiotic in MHB. These concentrations ranged from 0.125−3× the median MIC of the wild-type *S. aureus* ATCC 29213 clone. To meet the CLSI standard,^78^ we incubated the vancomycin and nafcillin cultures for 24 hours at 35°C. The other cultures were incubated at 37°C for 18 – 20 hours. The MIC of each sample was the lowest antibiotic concentration that inhibited visual growth.

We quantified the susceptibility of 3 technical replicates for each of the 18 evolved VISA populations across the 7 antibiotics, totaling 378 MIC measurements. In addition, we included the ancestor in each broth microdilution plate as a control and reference. This strain’s susceptibility was estimated with 8 technical replicates per drug, resulting in 56 MIC measurements. Collateral drug responses were calculated by log_2_(evolved MIC/ancestral MIC).

Then, we evaluated the likelihood (*L*) that a population will exhibit a particular drug response (collateral resistance or sensitivity) following vancomycin selection. We performed this analysis by comparing the collateral response of each replicate to the median response for all 18 replicates. If the responses had the same sign, we added 1*/N* to *L* (starting from *L* = 0), where *N* = 18 is the number of replicates. Otherwise, we did not add anything. The *L* estimates therefore range from 0.5, indicating that it is equally likely that any given population will be collaterally resistant or sensitive, to 1, indicating that a population will be one or the other in total agreement with the median.

## Data Availability

We have deposited the analysis code and bioinformatics data in GitHub (URL here). Sequence read data have been deposited in the National Center for Biotechnology Information Sequence Read Archive (URL here) (accession no: xxx). Accession no. for the ATCC 29213 strain is LHUS00000000.
