# Supplementary material for: Evolution under vancomycin selection drives divergent collateral sensitivity patterns in Staphylococcus aureus
Source: bioRxiv. 2025 Apr 5:2023.11.30.569373. Originally published 2023 Dec 1. Preprint. [Version 3] doi: 10.1101/2023.11.30.569373 (PMC10705500; doi:10.1101/2023.11.30.569373)
Supplement: Supplement 1 [file NIHPP2023.11.30.569373v3-supplement-1.pdf]

bioRxiv preprint doi: https://doi.org/10.1101/2023.11.30.569373; this version posted April 5, 2025. The copyright holder for this preprint (which was not certified by peer review) is the author/funder, who has granted bioRxiv a license to display the preprint in perpetuity. It is made available under aCC-BY 4.0 International license.

SI Methods

Following the feature selection by multivariate logistic regression, we performed Bayesian latent class analysis (BLCA) using the R package BayesLCA (1) with selected features (genes) mutated in at least three lines to identify distinct evolutionary trajectories. In this framework, the posterior distribution of parameters given the observations can be written as,

$$\prod^{N}\prod^{G}\tau^{z_{ig}+\delta_{g}-1}\prod^{M}\theta_{gm}^{x_{im}z_{ig}+\alpha_{gm}-1}(1-\theta_{m})^{(1-x_{im})z_{ig}+\beta_{gm}-1}$$

where τ is the estimate for the population frequency of a particular group, θ is a GxM dimensional matrix of mutation probability given the group assignment, and δ, α, and β are parameters for the conjugate prior distributions for the Dirichlet and Beta distributions, respectively. We set the δ parameter to 1.0 as a uniform prior over class distributions and α and β to 0.5 to increase density around 0.0 and 1.0. Given the computational constraints of Markov chain Monte Carlo-based estimation, we used the expectation-maximization algorithm. To further improve the robustness of the clustering, we used the BLCA over 1000 iterations, where a random 90% subsampling was performed for both features and observations at each iteration. The subsampled data was clustered with k set from 1 to 5, and the best number of clusters was selected using AIC. At each clustering iteration, we tracked which observations were clustered together. Subsequently, we created a consensus matrix quantifying the frequency of pairwise observations, and a final clustering was performed using hierarchical clustering with Ward's criteria on the frequency matrix.

bioRxiv preprint doi: https://doi.org/10.1101/2023.11.30.569373; this version posted April 5, 2025. The copyright holder for this preprint (which was not certified by peer review) is the author/funder, who has granted bioRxiv a license to display the preprint in perpetuity. It is made available under aCC-BY 4.0 International license.

SI Figures

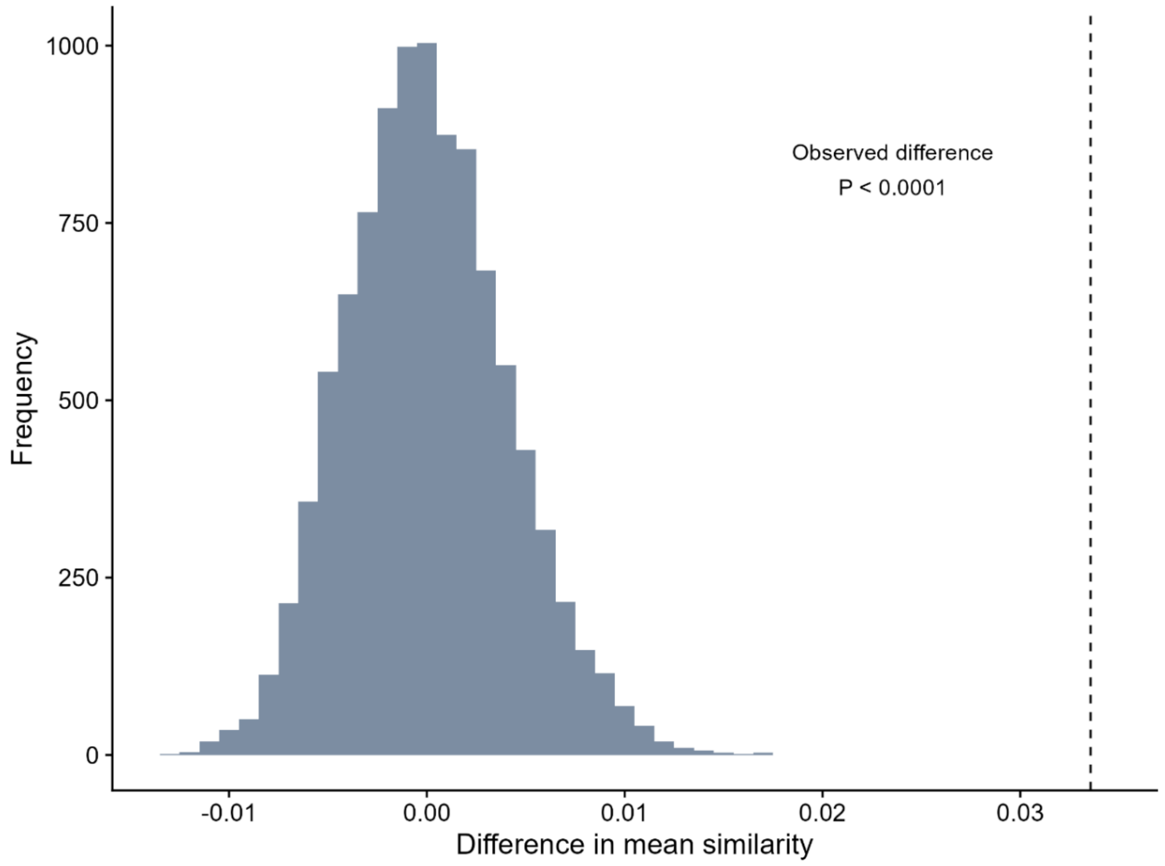

Fig. S1. The null distribution of the difference in mean gene-level similarity between vancomycin-adapted and control populations. We calculated Dice's similarity coefficients for each population pair using only the qualifying mutations that unambiguously affected single genes. We assessed the difference in average similarity between the vancomycin-adapted and control lines. Then, we shuffled the population labels randomly between the two treatment groups and recalculated the difference in mean similarity. This process was repeated 10,000 times. The resulting distribution of differences is presented here. The observed difference of 0.034 surpassed all permutations (P < 0.0001), indicating a significantly higher level of genomic parallelism under vancomycin selection.

bioRxiv preprint doi: https://doi.org/10.1101/2023.11.30.569373; this version posted April 5, 2025. The copyright holder for this preprint (which was not certified by peer review) is the author/funder, who has granted bioRxiv a license to display the preprint in perpetuity. It is made available under aCC-BY 4.0 International license.

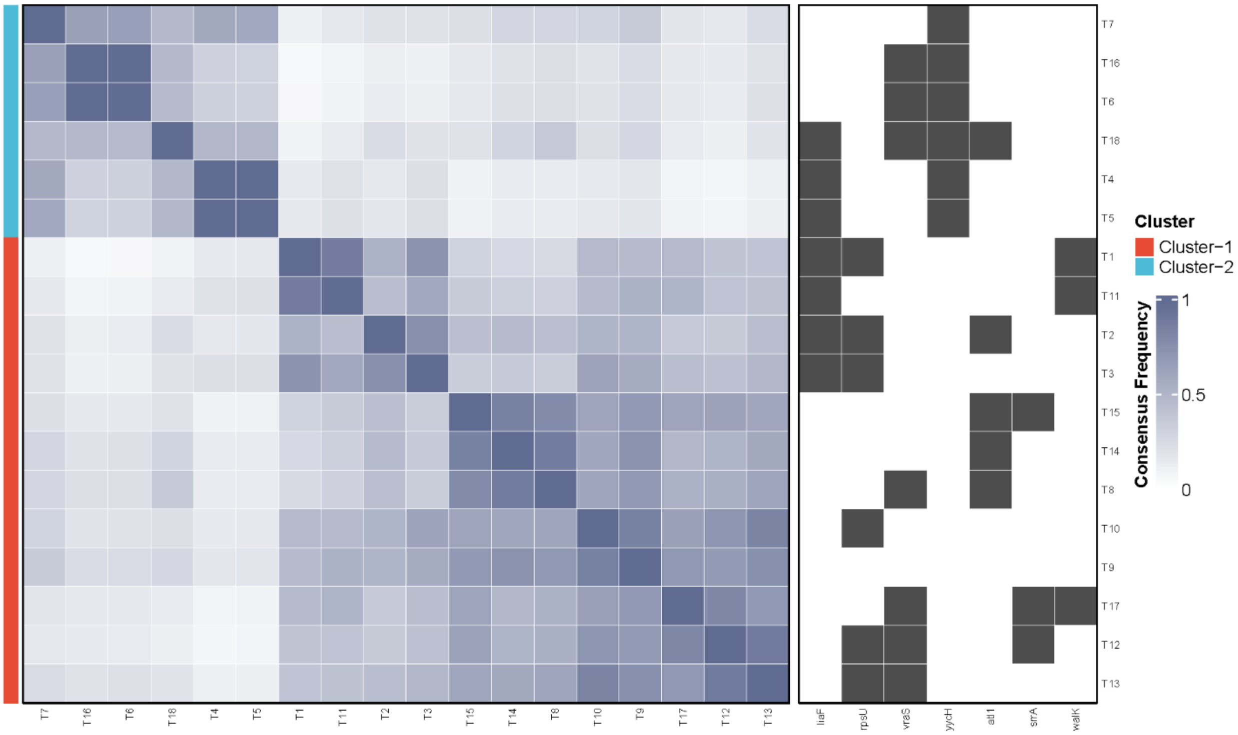

Fig. S2. Two distinct adaptive trajectories occurred under vancomycin selection. The heatmap represents the consensus matrix, generated through iterative Bayesian latent class analysis (BLCA) with random subsampling of observations and genomic features (Supplemental Methods), indicating the presence of two distinct clusters that correspond to unique genomic profiles.

bioRxiv preprint doi: https://doi.org/10.1101/2023.11.30.569373; this version posted April 5, 2025. The copyright holder for this preprint (which was not certified by peer review) is the author/funder, who has granted bioRxiv a license to display the preprint in perpetuity. It is made available under aCC-BY 4.0 International license.

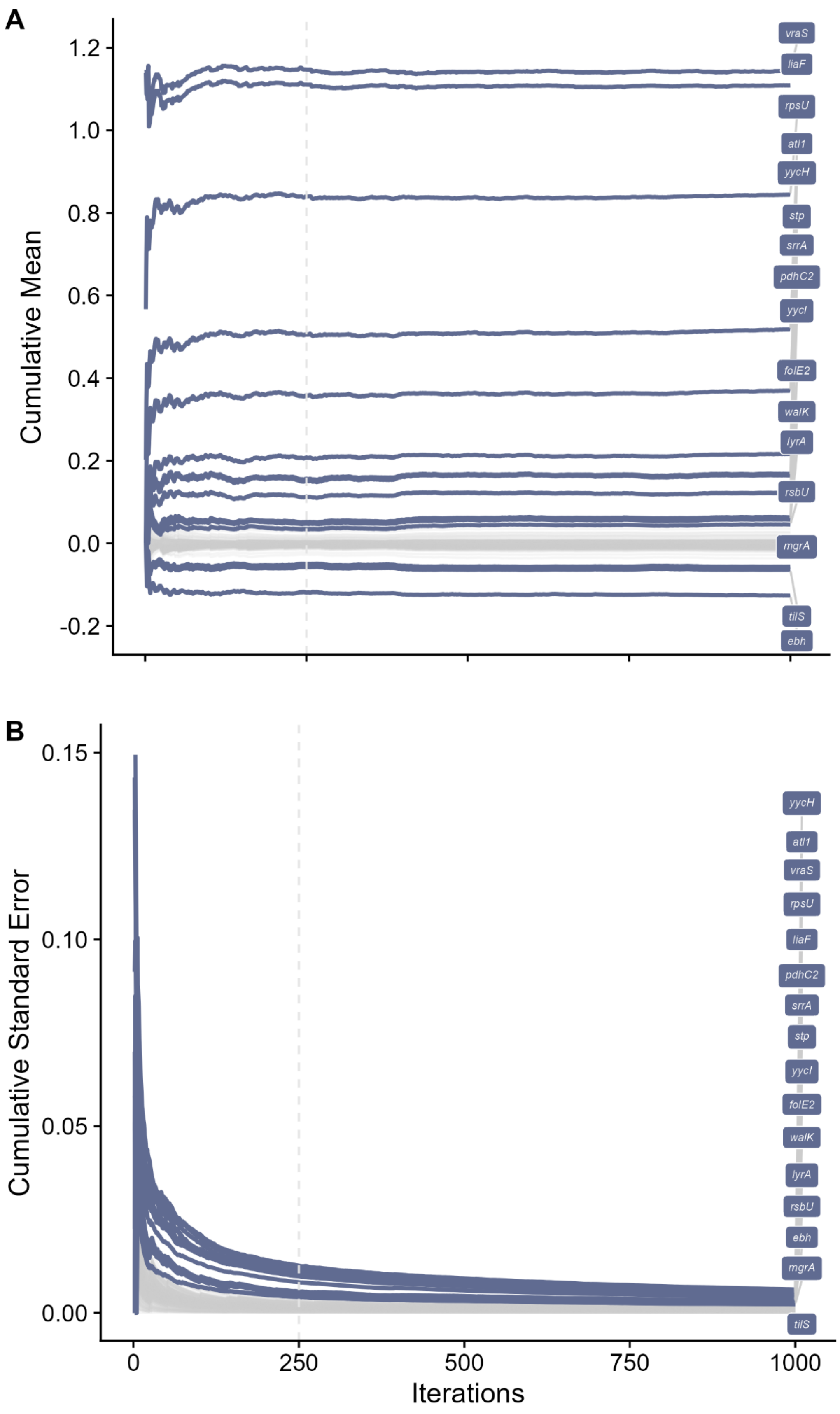

750

bioRxiv preprint doi: https://doi.org/10.1101/2023.11.30.569373; this version posted April 5, 2025. The copyright holder for this preprint (which was not certified by peer review) is the author/funder, who has granted bioRxiv a license to display the preprint in perpetuity. It is made available under aCC-BY 4.0 International license.

Fig. S3. Tests for convergence in logistic regression model coefficients. Over 1,000 iterations of a penalized regression procedure (Materials and Methods), we estimated (***A***) the cumulative mean and (***B***) the standard error for coefficients corresponding to qualifying gene-level mutations. Blue highlighted lines denote genes with non-zero coefficients in at least 20% of the iterations. Labels are ordered by decreasing cumulative mean and standard error. Grey highlighted lines indicate all other genes. The dotted line indicates the iteration where convergence is achieved.

bioRxiv preprint doi: https://doi.org/10.1101/2023.11.30.569373; this version posted April 5, 2025. The copyright holder for this preprint (which was not certified by peer review) is the author/funder, who has granted bioRxiv a license to display the preprint in perpetuity. It is made available under aCC-BY 4.0 International license.

SI Reference

1. A. White, T. B. Murphy, BayesLCA: An R package for Bayesian latent class analysis. *J. Stat. Softw.* **16**, 1–28 (2014).
